# Heterologous Expression of the *Lactobacillus sakei* Multiple Copper Oxidase to Degrade Histamine and Tyramine at Different Environmental Conditions

**DOI:** 10.3390/foods11203306

**Published:** 2022-10-21

**Authors:** Xiaofu Wang, Yunsong Zhao, Sufang Zhang, Xinping Lin, Huipeng Liang, Yingxi Chen, Chaofan Ji

**Affiliations:** 1 School of Food Science and Technology, Dalian Polytechnic University, Dalian 116034, China; 2 National Engineering Research Center of Seafood, Dalian 116034, China; 3 Department of Agricultural, Forest and Food Sciences, University of Torino, 10121-10156 Turin, Italy

**Keywords:** *Lactobacillus sakei*, biodegradation, multicopper oxidase, food matrices, biogenic amines

## Abstract

Biogenic amines (BAs) are produced by microbial decarboxylation in various foods. Histamine and tyramine are recognized as the most toxic of all BAs. Applying degrading amine enzymes such as multicopper oxidase (MCO) is considered an effective method to reduce BAs in food systems. This study analyzed the characterization of heterologously expressed MCO from *L. sakei* LS. Towards the typical substrate 2,2′-azino-bis (3-ethylbenzothiazoline-6-sulfonic acid) (ABTS), the optimal temperature and pH for recombinant MCO (rMCO) were 25 °C and 3.0, respectively, with the specific enzyme activity of 1.27 U/mg. Then, the effect of different environmental factors on the degrading activity of MCO towards two kinds of BAs was investigated. The degradation activity of rMCO is independent of exogenous copper and mediators. Additionally, the oxidation ability of rMCO was improved for histamine and tyramine with an increased NaCl concentration. Several food matrices could influence the amine-oxidizing activity of rMCO. Although the histamine-degrading activities of rMCO were affected, this enzyme reached a degradation rate of 28.1% in the presence of surimi. Grape juice improved the tyramine degradation activity of rMCO by up to 31.18%. These characteristics of rMCO indicate that this enzyme would be a good candidate for degrading toxic biogenic amines in food systems.

## 1. Introduction

Biogenic amines (BAs) are produced by microbial decarboxylation of free amino acids in various protein-rich foods, fermented foods, and beverages [1]. It is proven that consuming foods containing high amounts of BAs may cause toxicological reactions [2]. The most toxic BAs are histamine and tyramine, which also show synergistic toxicity [3]. Thus, the content of histamine and tyramine in foods must be controlled. The application of degrading amine enzymes is considered an effective method that demonstrates a more negligible effect on the nutrition and flavor of the food products [4].

Multicopper oxidases (MCO) exist in fungi, plants, and bacteria and catalyze the oxidation of many substrates, such as amines and aromatic compounds. Recently, MCO has received much attention due to its wide range of applications, such as degrading harmful substances and eco-friendliness (only releasing water as a byproduct) [5]. Several researchers have demonstrated the BAs degradation abilities of MCOs from species such as *Bacillus amyloliquefaciens* and *Enterococcus* spp. Given the importance of the application of MCOs, researchers should explore more novel enzymes from different species. Lactic acid bacteria (LAB) are essential sources of the BAs-degrading enzymes [6]. MCO from *Lactobacillus*, *Enterococcus*, or *Pediococcus* species can degrade different BAs [7,8]. Some MCOs degrade BAs in the presence of ABTS as the mediator [9]. However, the use of such enzymes in the food system is still limited because the food matrices are complex. MCOs should keep their ability at different environmental conditions, such as temperature, pH values, presence of inhibitors, etc.

Previously, a strain of *Lactobacillus sakei* LS was isolated from the fermented food and could degrade histamine and tyramine. This study aims to express the MCO from this *L. sakei* LS strain heterologously. The characteristics of expressed MCO were then investigated further, with the effects of ethanol, NaCl, and food matrix on MCO’s ability to degrade histamine and tyramine being investigated. This study will provide a potential candidate for the degradation of BAs in food systems.

## 2. Materials and Methods

### 2.1. Strains, Reagents, and Materials

*E. coli* DH5α and *E. coli* BL21 (DE3) were provided by Tiangen Biochemical Technology Co., Ltd. (Beijing, China). *Lactobacillus sakei* LS was isolated from a kind of fermented fish product [10]. TaKaRa MiniBEST Bacteria Genomic DNA Extraction Kit Ver.3.0, DNA Ligation Kit Ver.2.1, PrimeSTAR^®^ Max DNA Polymerase, enzymes (*SacI* and *XhoI*) and DNA markers for molecular cloning were obtained from Takara Bio Inc. (Dalian, China). Copper chloride (CuCl_2_), isopropyl-β-D-thiogalactopyranoside (IPTG), histamine, and tyramine were purchased from Aladdin Company (Shanghai, China). Nickel-chelating nitrilotriacetic acid (Ni-NTA) agarose gel was purchased from Shanghai Yuanye Biotechnology Co., Ltd. (Shanghai, China). Various kits (SanPrep Column Plasmid Mini-Preps Kit, SanPrep Column DNA Gel Extraction Kit, SanPrep Column PCR Product Purification Kit), 2,2 -azino-bis(3-ethylbenzothiazoline-6-sulfonic acid) (ABTS) were purchased from Sigma (Shanghai, China). Kanamycin was purchased from Shenggong Bioengineering Co., Ltd. (Shanghai, China). A protein marker was purchased from Biyuntian Biotechnology Co., Ltd. (Shanghai, China). LB medium was purchased from Qingdao Haibo Biotechnology Co., Ltd. (Qingdao, China). All other chemicals and reagents were analytical grade. Fish, tofu, and grape juice were purchased from the local market (Dalian, China).

### 2.2. Cloning and Heterologous Expression of the MCO Gene

PCR analysis was performed by amplifying the MCO gene of *Lactobacillus sakei* using the forward primer 5′-TTCGAGCTCATGAAAACCTATACGGACT-3′ and the reverse primer 5′-GTGCTCGAGCATTTTCATTCCCATTTT-3′. Recognition sites for *SacI* and *XhoI* endonucleases are indicated in underline. PCR was carried out using PrimeSTAR Max DNA polymerase under the following conditions: initial denaturation (95 °C for 3 min), 32 cycles of denaturation (95 °C for 0.5 min), primer annealing (55 °C for 0.5 min), and extension (72 °C for 1.5 min). Finally, reactions were completed with a 10 min elongation time at 72 °C followed by cooling to 4 °C. The product was purified by the SanPrep Column PCR Product Purification Kit following the manufacturer’s instructions. After double digestion with *SacI* and *XhoI*, the purified product was ligated into the linearized plasmid pET-28a to construct the recombinant plasmid pET-28a-LS. This recombinant plasmid was amplified in *E. coli* DH5α after heat shock transformation. After transforming pET-28a-LS into *E. coli* BL21 (DE3), the recombinant multicopper oxidases (rMCO) with a 6 × His-tag were heterologously expressed in *E. coli* BL21 (DE3). Briefly, positive *E. coli* transformed cells were cultured at 37 °C in LB medium supplemented with kanamycin (50 μg/mL) under shaking conditions (200 rpm). When the OD_600_ was 0.6–0.8, the culture was supplied with 0.1 mM IPTG and 1 mM CuCl_2_ (final concentration) to induce the expression of rMCO protein at 16 °C for 200 rpm for 20 h.

### 2.3. Purification of rMCO

The centrifugally (10,000× *g*, 4 °C, 10 min) collected cells were resuspended in 20 mM phosphate buffer (pH 7.0) containing 0.5 mg/mL lysozyme and kept on ice for 30 min. After that, the samples were repeatedly frozen and thawed three times to destroy the cells. The lysate was centrifuged (10,000× *g*, 4 °C, 10 min), and the supernatant was collected and loaded into the nickel-chelating nitrilotriacetic acid (Ni-NTA) agarose gel column. The column was previously equilibrated with the buffer solution (20 mM phosphate, 500 mM NaCl, 20 mM imidazole, pH 7.4). Then, rMCO was eluted with an imidazole solution (20 mM phosphate, 500 mM NaCl, 300–500 mM imidazole, pH 7.4). The fractions were collected and ultrafiltered. The molecular weight and purity of rMCO were analyzed by sodium dodecyl sulfate polyacrylamide gel electrophoresis (SDS-PAGE). With bovine serum protein as the standard, the protein concentration of the sample was determined by the Bradford method.

### 2.4. rMCO Activity Assay

The enzyme activity of rMCO was determined according to a previously published protocol [11]. In brief, the samples were mixed with 0.1 M citrate-phosphate buffer (pH 3) containing 0.5 mM ABTS and incubated at 25 °C for 3 min. ABTS oxidation was determined by the change of absorbance at 420 nm (ε_420_ = 36,000 M^−1^ cm^−1^). One unit of enzyme activity was defined as the amount of enzyme required to oxidize 1 μmol ABTS per minute under the above conditions.

ABTS was used as a substrate in the following characterization experiments. The enzymatic reaction was performed in 0.1 M citrate-phosphate buffer (pH 2.2–8.0) and glycine-sodium hydroxide buffer (pH 9.0–10.0) to determine the optimal reaction pH. The optimum reaction temperature was evaluated from 20 to 90 °C. The relative enzyme activity was calculated with a maximum enzyme activity of 100%. The effect of metal ions, potential inhibitors, and organic reagents on the activity of rMCO was assayed by determining the relative activity of the enzyme in the reaction system containing individual effectors. The enzyme activity measured without any of the effectors was considered 100%.

### 2.5. Amine-Oxidizing Activity of rMCO

The catalytic activity of rMCO towards histamine and tyramine under different conditions was studied at 25 °C for 24 h. In the reaction system, the concentration of histamine and tyramine was 100 mg/L. Different enzymatic reaction conditions include enzyme concentration (7, 17, 33, 66, and 132 U/L, pH 7), ethanol concentration (0, 10, 15, 20%, *v/v*, pH 7), and NaCl concentration (0, 2, 4, 6, 8, 10%, m/v, pH 4 and 7). Except for the experiment evaluating enzyme concentration dependence, the dose of enzyme added to other reactions was 33 U/L.

The amine-oxidizing activity of rMCO in the food matrix system was further investigated. The reaction system containing surimi (pH 7) was 0.16 g/mL mackerel surimi, 100 U/L rMCO, 200 mg/L histamine, 50 mg/L tyramine, and 6% NaCl. The mixture reacted at 15 °C for 24 h. The reaction system containing tofu (pH 7) contained 0.16 g/mL tofu surimi, 100 U/L rMCO, 200 mg/L histamine, 200 mg/L tyramine, and 6% NaCl. The mixture reacted at 30 °C for 24 h. The third reaction system (pH 4) was comprised of 26% (*v/v*) pure grape juice, 100 U/L rMCO, 50 mg/L histamine, 50 mg/L tyramine, and 10% ethanol. The mixture reacted at 30 °C for 24 h.

After the reaction, the residual amine concentration was detected by the ultra-performance liquid chromatography-mass spectrometry (UPLC-MS) method [12]. The reaction mixtures without enzymes under the same conditions were used as negative controls. All experiments were repeated three times.

## 3. Results and Discussion

### 3.1. Sequence Analysis and Heterologous Expression of the rMCO Gene from L. sakei Ls

**Figure 1:**
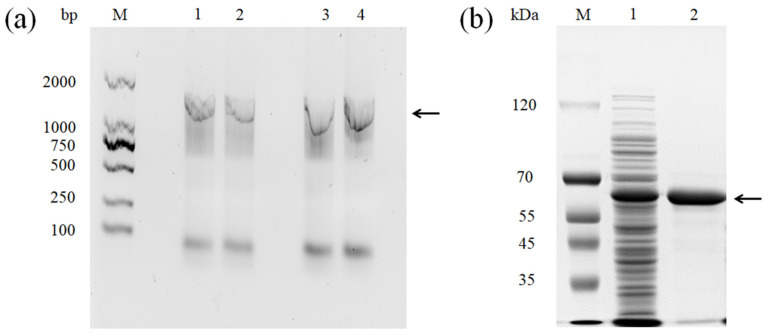
(**a**) Multicopper oxidase (MCO) gene amplification. Lane M, molecular weight markers (100–2000 bp); lanes 1–2: PCR amplification of *L. sakei* Ls to detect genes encoding MCOs; and lanes 3–4: PCR amplification of recombinant *E. coli* BL21(DE3) to detect genes encoding MCOs. (**b**) Analysis of the molecular weight of expressed and purified rMCO by SDS-PAGE (Lane M: protein marker; Lane 1: crude protein of *E. coli* BL21 lysate; Lane 2: purified rMCO. Black arrow: band of rMCO).

Using BAs-degrading enzymes is a promising method to reduce BAs content in food systems. In a previous study, our lab isolated a BAs degrading strain, *L. sakei* LS, from fermented fish products [10]. The gene encoding MCO was amplified with the genome of *L. sakei* as the template (Figure 1a). Sequence analysis indicated the length of the PCR product consisted of 1530 nucleotides, encoding a protein containing 510 amino acids with a predicted molecular weight of 58 kDa. Then, the MCO gene was cloned into the pET-28a expression vector and transformed into *E. coli* BL21 (DE3). SDS-PAGE analysis of *E. coli* BL21 cell lysates and the purified recombinant MCO (rMCO) are presented in Figure 1b. The rMCO was purified to homogeneity, exhibiting a single band. The purified protein showed a blue color in the solution, similar to other MCOs [7].

**Figure 2:**
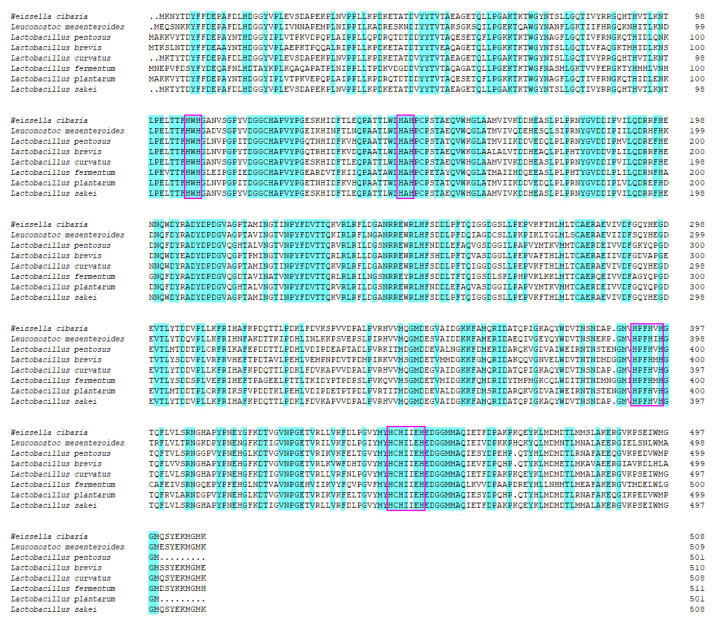
Amino acid sequence alignment of rMCO and other MCOs from different LAB species. Identical amino acids are highlighted in solid blue. Encased inside boxes represent the highly conserved motifs forming four copper ligands in MCOs.

The sequence of MCO from *L. sakei* LS displayed a high homology with the MCOs from other lactic acid bacteria (LAB) species, showing about 80.88% identity at the amino acid level (Figure 2). The MCO from *L. sakei* contains four copper ligand motifs, HXH, HXH, HXXHXH, and HCHXXXH, which are highly conserved in the MCO family [13]. The fourth motif is related to the enzyme active site T1Cu responsible for the preliminary oxidation of substrates, in which the function of the Cys445 residue is critical.

**Figure 3:**
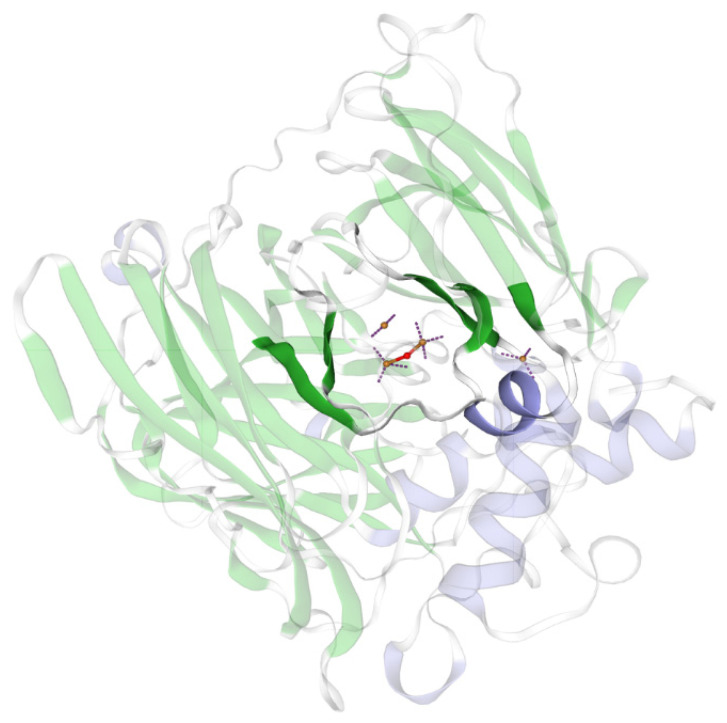
Predicted 3D structure model of the rMCO. The three copper centers are highlighted. The yellow balls stand for Cu^2+^ in the active site.

The predicted 3D structure of rMCO is presented in Figure 3, using the MCO from *Pediococcus pentosaceu* as a template for homology modeling. The monomeric rMCO contains three cupredoxin-like domains. These results indicate that this study’s recombinant enzyme heterologously expressed is a typical MCO that could potentially degrade biogenic amines.

### 3.2. Enzymatic Characterization of rMCO

**Figure 4:**
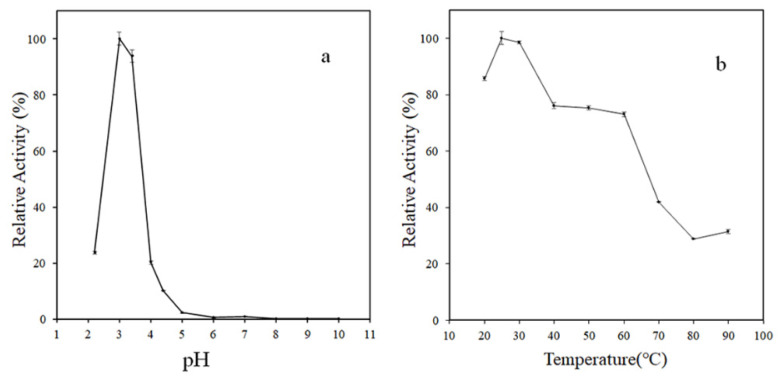
Effect of temperature (**a**) and pH (**b**) on the activity of rMCO towards ABTS.

ABTS was used as the typical substrate to evaluate the enzymatic characteristics of MCO. In this study, the effect of pH and temperature on the catalytic activity of rMCO towards ABTS was investigated and presented in Figure 4. The highest oxidation activity of rMCO was found at pH 3.0 and 25 °C. Under this optimum condition, the specific activity of the purified rMCO was around 1.27 U/mg. The optimum condition was different from other bacterial MCOs. For example, the laccase from *Bacillus velezensis* TCCC 111904 exhibited maximal activity at pH 5.5 and 80 °C [11]. The laccase from *Fusarium oxysporum* HUIB02 exhibited maximal activity at pH 3.5 and a temperature of 40 °C [14]. Overall, the rMCO exhibited high activity under acidic conditions. Furthermore, it retained good catalytic ability over a wide temperature range from 20 to 60 °C. The rMCO has potential applications in acidic food processing.

Afterwards, the effects of some metal ions, enzyme inhibitors, and organic solvents on the activities of rMCO were analyzed and presented in Table 1. Among all the metal ions, Fe^2+^ and Fe^3+^ show a pronounced inhibition effect on the activity of rMCO. Significantly, 5 mM Fe^2+^ reduced the activity of rMCO to about 2.2%. In contrast, the enzymatic activity of rMCO remained above 65% when the concentration of other metal ions increased to 5 mM. Interestingly, 0.5 mM Mg^2+^ even slightly enhanced the activity of rMCO. Although MCOs from different species demonstrate remarkably different tolerance against various metal ions, ferric ions often negatively affect enzyme activity [15]. Cu^2+^ is essential in the MCO synthesis, while the MCO dependence from different strains on exogenous copper varied. For example, copper dependence was observed in CueO from *Escherichia coli* but not in CotA from *Bacillus subtilis* [16]. In this study, adding exogenous copper did not significantly contribute to the rMCO activity. The independence of exogenous copper for the rMCO indicated that this enzyme would be a good candidate for the application in food systems. The effects of other potential inhibitors and organic solvents on rMCO activity are also shown in Table 1. The enzymatic activity of rMCO was severely inhibited by SDS and L-Cysteine at a low concentration, but not by EDTA. The reason that EDTA has little effect on the enzyme activity may be related to the stability of the copper center of the enzyme [7]. Furthermore, the rMCO can maintain relatively high enzymatic activity in the presence of the 1% organic solvent listed in Table 1. Additionally, it can still retain about 70.6% of its activity in 10% ethanol, indicating that rMCO could work in wines.

### 3.3. Amine-Oxidizing Activity of rMCO

**Figure 5:**
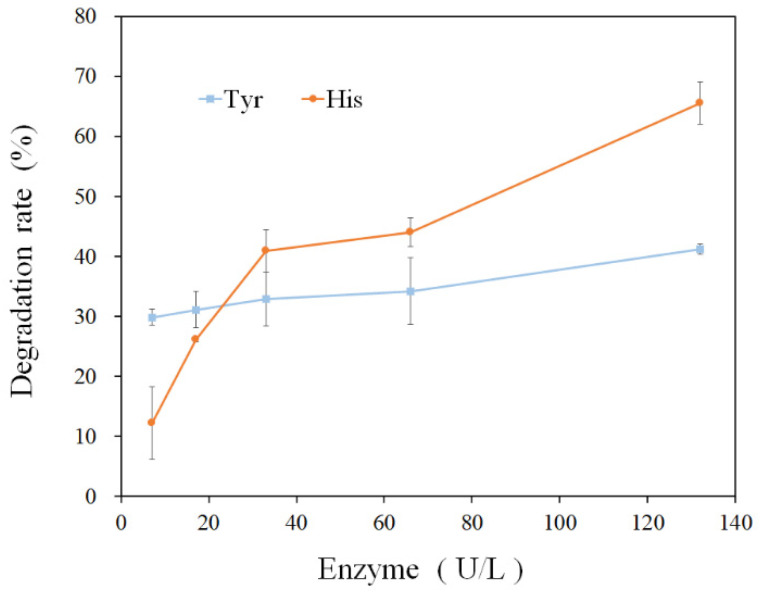
Histamine and tyramine degradation rates of rMCO.

Histamine and tyramine are the two most toxic biogenic amines [17]. Therefore, the amine-oxidizing activity of rMCO towards histamine and tyramine was studied. Firstly, the degradation rates of histamine and tyramine increased with the concentration of rMCO (Figure 5). The dependence of histamine on enzyme concentration was higher compared to tyramine. When the concentration of rMCO increased, tyramine and histamine degradation rates reached 41.24% and 65.5%, respectively. The catalytic ability of MCO towards substrates is related to the substrate’s phenolic structure [18]. Although some MCOs from LAB degrade tyramine only in the presence of the mediator ABTS [9], it is noteworthy that the rMCO can degrade histamine and tyramine without mediators.

**Figure 6:**
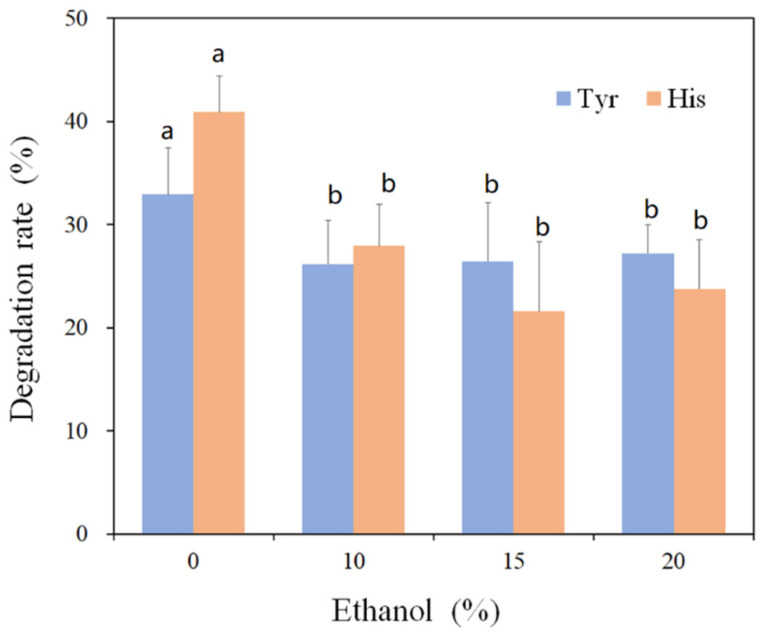
Effect of ethanol content on the activity of rMCO towards histamine and tyramine. Different letters indicate significantly different (*p* < 0.05) mean values.

The histamine- and tyramine-oxidizing activities of rMCO in the presence of ethanol are explored. As shown in Figure 6, the efficiency of tyramine and histamine degradation decreases as ethanol concentration increases. High organic solvent concentration typically suppresses enzyme activity because the active catalytic conformation of the enzyme is disrupted to some extent [19]. It is worth noting that when the ethanol concentration is increased to 20%, rMCO can maintain 58.1% histamine-oxidizing activity and 82.64% tyramine-oxidizing activity. The 20% ethanol concentration is higher than that of alcoholic beverages, which varies between 12 and 16% [20]. As a result, rMCO has the potential to be used to degrade BAs in alcohol products.

**Figure 7:**
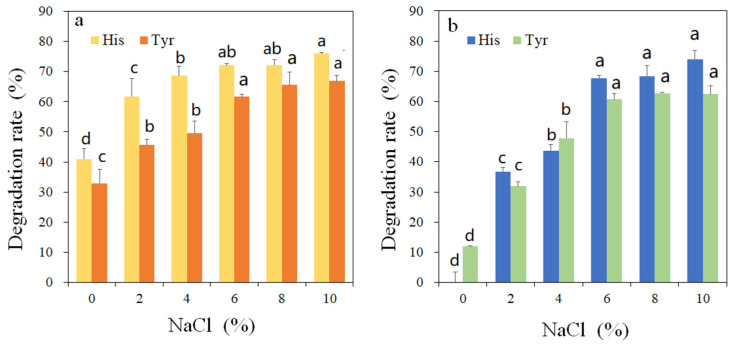
Effect of NaCl on the activity of rMCO towards histamine and tyramin. (**a**) pH = 7 and (**b**) pH = 4. Different letters indicate significantly different (*p* < 0.05) mean values.

Sodium salt is the most frequently used ingredient in protein-rich fermented products that may contain a high level of BAs. Since highly concentrated NaCl is likely to inhibit the enzyme activity, the effect of NaCl content on the amine-oxidizing efficiency of rMCO was investigated in acidic and neutral environments. The variation in decomposition rate is generated by the redox potential difference between a reducing substrate and the type 1 copper of laccase (which correlates to the electron transfer rate and is favored for a phenolic substrate by higher pH) [21]. As presented in Figure 7, the rMCO demonstrated higher oxidation efficiency of histamine and tyramine in the neutral environment than in the acidic environment. The optimum pH for BAs oxidation is inconsistent with the optimal pH for ABTS oxidation, indicating that the optimal pH of rMCO is substrate dependent. This phenomenon is because the conformation of enzyme proteins and the redox potential of the enzyme will change due to the influence of the pH environment [21]. Meanwhile, the optimal pH value of rMCO for the oxidation of BAs is also different from that of *Lactobacillus plantarum*, and the tyramine degradation rate of the latter reaches its peak at pH 4 [7]. Even if highly conserved active sites are shared, the substrate ranges and catalytic activity of enzymes from different species are quite different. This results from a combination of factors, including partial structural features, the interaction of copper ligands, and the restriction of protein folding [22]. The oxidation ability of rMCO was improved for histamine and tyramine with the increased NaCl concentration, which benefited from salt activation. The salt activation of multicopper oxidases was substrate dependent. Furthermore, the MCOs from different sources have remarkably different levels of tolerance towards NaCl [23]. The high salinity tolerance of rMCO would be advantageous in treating various food systems that usually contain a high concentration of NaCl.

### 3.4. Effect of Food Matrix on the Amine-Oxidizing Activity of rMCO

**Figure 8:**
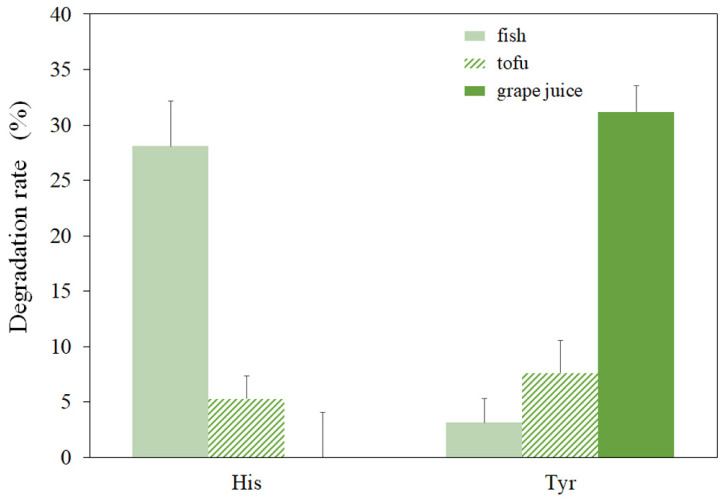
Effect of food matrices on the activity of rMCO towards histamine and tyramine.

In the previous section, rMCO performs well in degrading histamine and tyramine in the buffer solution. However, the components in food are much more complex than those in buffer solutions. Since the substrate range of MCO is broad, the substrate selectivity of MCOs should be concerned with its application in food. Here, the effect of the food matrix on the amine-oxidizing activity of rMCO was analyzed. Since BAs were mainly detected in fermented foods, the histamine- and tyramine-oxidizing activities of rMCO were determined by adding fish surimi, tofu, and grape juice to the catalytic solutions. As shown in Figure 8, the highest histamine degradation rate of 28.1% is observed in the catalytic system containing fish surimi, while the tyramine degradation rate is as low as 3.15%.

On the contrary, histamine was not degraded, and the tyramine degradation rate was 31.18% in the system with added grape juice. It is worth mentioning that histamine is the most abundant and harmful BA in fermented fish products. The histamine oxidizing ability of rMCO in fish surimi-containing systems proves that it has the value of application in the fish production industry. Interestingly, the tyramine degradation efficiency of rMCO in the grape juice system was better than that in the buffer solution. Free amino acids and small molecular peptides in fish surimi are also potential MCO substrates, so the competition may lead to the dispersion of enzyme activity [24]. The enhanced efficiency of rMCO may be due to the small-molecular phenols in grape juice, such as proanthocyanidins, chlorogenic acid, epicatechin, and gallic acid [25,26]. These small-molecular phenols can act as mediators to aid rMCO in improving amine oxidation efficiency [5,27]. The tannic acid rich in wine may become an obstacle to the practical application of rMCO. They may interfere with substrate reduction or inhibit rMCO activity [28,29]. The degradation rate of both amines in the tofu system is less than 10%, which may be related to monoamine oxidase inhibitory peptides in tofu [30]. There may also be higher levels of calcium and magnesium ions that may also have adverse effects [31]. The amine-oxidizing activity of rMCO is not ideal in the simulated fermentation environment with a food matrix, but it still shows the potential of the application. Future scientific research should be carried out to improve the in-situ catalytic degradation of BAs with MCOs in food systems. In addition, in future commercial applications, strains such as yeast and *Bacillus subtilis* could be chosen to heterologously express MCOs.

## 4. Conclusions

LAB species are good sources of BA-degrading enzymes, such as MCOs, since this type of bacteria could reduce BAs in different foods. In this study, the MCO gene of *Lactobacillus sakei* was cloned and heterologously expressed. The optimal pH for catalytic efficiency by the enzyme is related to the type of substrate: an acidic environment is required to catalyze the typical substrate ABTS, and histamine and tyramine are catalyzed with high efficiency in a neutral environment without mediators. This study first demonstrated that the type of food matrix significantly affects the catalytic activity of this enzyme, because MCO is capable of oxidizing a wide spectrum of substrates. Thus, there are probably many competitive inhibitors in food. Additionally, food matrices rich in natural mediators could facilitate the oxidative reactions. The design of tailor-made MCOs for different foods will result in high-efficiency applications and lead to a deep understanding of the catalytic mechanism.

## Figures and Tables

**Table 1:** Effects of metal ions, inhibitors, or organic reagents on the activity of rMCO. Values are means ± standard deviations of triplicate assays.

Metal Ions /Inhibitors /Organic Reagents	Concentration (mM/%)	Relative Activity (%)
None	-	100 ± 2.4
CuCl_2_	0.5	95.7 ± 2.4
	5	68.1 ± 1.7
MgCl_2_	0.5	101.5 ± 3.4
	5	75.9 ± 2.0
FeCl_2_	0.5	11.1 ± 0.8
	5	2.2 ± 0.1
MnSO_4_	0.5	98.9 ± 2.7
	5	103.1 ± 1.9
ZnCl_2_	0.5	95.9 ± 2.3
	5	80.1 ± 1.5
KCl	0.5	101.2 ± 2.0
	5	85.4 ± 2.1
CaCl_2_	0.5	98.8 ± 2.9
	5	72.5 ± 1.8
FeCl_3_	0.5	96.6 ± 2.0
	5	51.0 ± 1.2
NaCl	0.5	96.5 ± 2.9
	5	79.3 ± 2.1
SDS	0.5	1.4 ± 0.1
	5	0.3 ± 0.1
EDTA	0.5	100.0 ± 2.6
	5	96.8 ± 2.9
L-Cysteine	0.5	22.2 ± 0.7
	5	0.4 ± 0.1
Methanol	1%	94.4 ± 2.0
	10%	80.6 ± 2.2
Ethanol	1%	97.2 ± 1.6
	10%	70.6 ± 1.9
Isopropanol	1%	98.2 ± 2.3
	10%	61.8 ± 2.5
Acetonitrile	1%	85.3 ± 1.9
	10%	24.9 ± 0.6
Acetone	1%	101.9 ± 2.9
	10%	41.2 ± 1.0

The concentration of organic solvent is the volume ratio. “-” means no metal ions, inhibitors, or organic reagents added.

## Data Availability

The data that support the findings of this study are available from the corresponding author upon reasonable request.
